# Repetitive Transcranial Magnetic Stimulation of the Dorsolateral Prefrontal Cortex Modulates Electroencephalographic Functional Connectivity in Alzheimer’s Disease

**DOI:** 10.3389/fnagi.2021.679585

**Published:** 2021-07-07

**Authors:** Yi Guo, Ge Dang, Brenton Hordacre, Xiaolin Su, Nan Yan, Siyan Chen, Huixia Ren, Xue Shi, Min Cai, Sirui Zhang, Xiaoyong Lan

**Affiliations:** ^1^Department of Neurology, Shenzhen People’s Hospital (The Second Clinical Medical College, Jinan University, The First Affiliated Hospital, Southern University of Science and Technology), Shenzhen, China; ^2^Shenzhen Bay Laboratory, Shenzhen, China; ^3^Innovation, Implementation and Clinical Translation (IIMPACT) in Health, Allied Health and Human Performance, University of South Australia, Adelaide, SA, Australia; ^4^CAS Key Laboratory of Human-Machine Intelligence-Synergy Systems, Shenzhen Institutes of Advanced Technology, Chinese Academy of Sciences, Shenzhen, China

**Keywords:** repetitive transcranial magnetic stimulation, electroencephalographic functional connectivity, power envelope connectivity, Alzheimer’s disease, cognitive function

## Abstract

**Background:** Increasing evidence demonstrates that repetitive transcranial magnetic stimulation (rTMS) treatment of the dorsolateral prefrontal cortex is beneficial for improving cognitive function in patients with Alzheimer’s disease (AD); however, the underlying mechanism of its therapeutic effect remains unclear.

**Objectives/Hypothesis:** The aim of this study was to investigate the impact of rTMS to the dorsolateral prefrontal cortex on functional connectivity along with treatment response in AD patients with different severity of cognitive impairment.

**Methods:** We conducted a 2-week treatment course of 10-Hz rTMS over the left dorsolateral prefrontal cortex in 23 patients with AD who were split into the mild or moderate cognitive impairment subgroup. Resting state electroencephalography and general cognition was assessed before and after rTMS. Power envelope connectivity was used to calculate functional connectivity at the source level. The functional connectivity of AD patients and 11 cognitively normal individuals was compared.

**Results:** Power envelope connectivity was higher in the delta and theta bands but lower in the beta band in the moderate cognitive impairment group, compared to the cognitively normal controls, at baseline (*p* < 0.05). The mild cognitive impairment group had no significant abnormities. Montreal Cognitive Assessment scores improved after rTMS in the moderate and mild cognitive impairment groups. Power envelope connectivity in the beta band post-rTMS was increased in the moderate group (*p* < 0.05) but not in the mild group. No significant changes in the delta and theta band were found after rTMS in both the moderate and mild group.

**Conclusion:** High-frequency rTMS to the dorsolateral prefrontal cortex modulates electroencephalographic functional connectivity while improving cognitive function in patients with AD. Increased beta connectivity may have an important mechanistic role in rTMS therapeutic effects.

## Introduction

Alzheimer’s disease (AD) is an irreversible neurodegenerative disorder that progressively destroys memory, language, other thinking abilities, as well as motor skills such as balance and mobility (Voglein et al., 2019; Alzheimer’s Association, 2021). Several randomized controlled studies have advocated using repetitive transcranial magnetic stimulation (rTMS) applied to the left dorsolateral prefrontal cortex (DLPFC) as a potential intervention to improve cognitive function in patients with Alzheimer’s disease (AD) or its possible precursor, mild cognitive impairment (MCI) (Cotelli et al., 2006; Ahmed et al., 2012; Rabey et al., 2013). Furthermore, considering the link between cognition and motor behavior, with evidence that stable gait relies on intact cognition, rTMS therapy might be an innovative tool for falls prevention in AD (Amboni et al., 2013). Nevertheless, the understanding of the mechanism(s) by which rTMS exerts its effects of improving cognition is incomplete. Animal models have shown that magnetic stimulation improves cognitive performance by attenuating the Aβ load, strengthening synaptic function, or increasing neurogenesis (Ueyama et al., 2011; Ma et al., 2017; Zhen et al., 2017). These rTMS-induced molecular and cellular alterations may act as the substrate of the brain network level changes, which have been more thoroughly studied in humans (Tik et al., 2017). rTMS evidently results in clinical improvement partially by modifying brain connectivity in patients with neuropsychological diseases such as stroke, Parkinson’s disease, and major depressive disorder (Beynel et al., 2020). Given that disrupted functional connectivity is a feature of AD (Briels et al., 2020a; Franzmeier et al., 2020; Smailovic et al., 2020), this neural mechanism may underpin rTMS treatment and is therefore worthy of further investigation.

Electroencephalography (EEG) is a neurophysiologic technique for evaluating brain activity. It can capture neural activity with high temporal resolution and no notable adverse effects. This low-cost technology is readily available in clinical settings. A recent white paper has advocated using EEG as a multiscale methodology for detecting the effects of neuropathological processes and the effects of therapy on brain activity and connectivity in preclinical and clinical research (Babiloni et al., 2020). Investigators have most frequently reported that delta and theta power density is increased across the whole brain, while alpha and/or beta powers are decreased in the posterior head in patients with AD, compared to normal individuals (Babiloni et al., 2016; Gouw et al., 2017). Distinct from power density, functional connectivity analysis has revealed impaired communication within long-range brain networks, and EEG connectivity data have strongly supported the hypothesis of AD as a disconnection syndrome (Delbeuck et al., 2003). Most previous studies have found decreased coherence of alpha and beta rhythms globally or locally in people with AD, compared to normal controls. However, data supporting alterations in delta and theta coherence are contradictory, with a widespread or regional increase or decrease being reported in different studies (Jeong, 2004; Babiloni et al., 2016).

Of note, traditional EEG connectivity metrics such as coherence have limitations because they do not account for the limited spatial resolution of EEG and volume conduction, which can result in an inaccurate interpretation of connectivity (He et al., 2019). Power envelope connectivity (PEC) is a novel method that biases against signals with zero phase-lag through orthogonalization to reduce the effects of volume conduction. As a result, PEC provides a more conservative estimate of functional connectivity. PEC has been introduced for addressing this artifactual correlation in magnetoencephalography (MEG) and EEG functional connectivity analysis (Hipp et al., 2012). In an MEG study (Koelewijn et al., 2017), orthogonalized amplitude envelope correlation (which is similar to PEC) in the source-space was compared between AD patients and controls. The patients had decreased alpha connectivity in the left superior temporal pole and disruption of the beta band in the right lingual gyrus, precuneus, and left supramarginal gyrus. A recent AD clinical trial found that, compared to a placebo, a novel glutaminyl-cyclase inhibitor increased the channel-level orthogonalized amplitude envelope correlation in the alpha band globally, which suggested the drug treatment had a network level effect (Briels et al., 2020b) and may serve as a mechanism of rTMS efficacy.

Some reports indicate a correlation between functional connectivity and severity of cognitive decline in AD (Hata et al., 2016; Badhwar et al., 2017) and the probable different effects of rTMS in patients at different stages of cognitive dysfunction (Cotelli et al., 2008). Therefore, the purpose of this study was to investigate how rTMS to the left DLPFC exerts its effects on cognitive enhancement in AD patients with different severity of cognitive decline. We hypothesized that left DLPFC rTMS would improve cognitive function in AD patients with mild or moderate cognitive impairment, at least in part, by changing the electroencephalographic functional connectivity in different bands, globally or locally.

## Materials and Methods

### Study Participants

This study was approved by the Institutional Review Board of Shenzhen People’s Hospital (Shenzhen, China) and was conducted in accordance with the Declaration of Helsinki guidelines (chictr.org.cn identifier ChiCTR1800019199). All participants provided written informed consent. From March 2019 to May 2020, 23 patients were recruited from the Department of Neurology in Shenzhen People’s Hospital. For selecting patients, the inclusion criteria were as follows: (a) meeting the diagnostic criteria for probable AD based on the National Institute of Neurological and Communicative Disorders and Stroke and the Alzheimer’s Disease and Related Disorders Association (NINCDS-ADRDA), and the Diagnostic and Statistical Manual of Mental Disorders-V (DSM-V) criteria; (b) no plan to change medication during the study and for at least 2 months prior to starting the study; (c) 50–75 years old; and (d) self-reported right-handedness. The exclusion criteria were: (a) severe cognitive impairment as defined by a score of < 10 on the Montreal Cognitive Assessment (MoCA)^1^; (b) severe depression as determined by a score >20 on the 24-item Hamilton Depression Rating Scale (HAMD) (Carrozzino et al., 2020); (c) a history of other psychiatric or other neurological disorders, such as schizophrenia, Parkinson’s disease, and multiple sclerosis; (d) a history of brain injury or cranial neurosurgery; (e) a history of alcohol or drug abuse; (f) any severe chronic systemic illness, such as heart failure and renal failure; and (g) any contraindication for rTMS (Rossi et al., 2009), such as a metallic implanted device in or near the head and aneurysms. Patients were divided into two groups based on the median split of their Montreal Cognitive Assessment (MoCA) score (i.e., 18/30) (Nasreddine et al., 2005). Twelve patients with a MoCA score ≥ 18 were grouped as having mild cognitive impairment (i.e., mild group), and 11 patients with a MoCA score < 18 were grouped as having moderate cognitive impairment (i.e., moderate group). Twelve healthy subjects who had never complained of cognition problems were recruited (i.e., control group). All of them met the following inclusion criteria: Aged 50–75 years old, self-reported right handedness, no family history of dementia, and no history of any psychiatric or neurological disorders, brain injury, cranial neurosurgery, alcohol or drug abuse, or any severe chronic systemic illness. Demographic and clinical data of the patients and controls are reported in Table 1.

**TABLE 1:** Demographic and clinical data.

	**Control**	**Mild**	**Moderate**
N	11	12	11
Age (years)	63.7 ± 2.0	65.8 ± 8.4	70.2 ± 5.8
Sex (male/female)	3/9	5/7	4/7
Education (years)	10 ± 5.7	11.1 ± 5.2	11.7 ± 3.3
RMT (%)	/	31.6 ± 9.7	30.4 ± 9
MoCA (baseline)	29.2 ± 1.1	23.2 ± 3.7	13.4 ± 2
HAMD (baseline)	4.6 ± 2.5	9.8 ± 4	9.9 ± 6.1

### Transcranial Magnetic Stimulation

All patients underwent rTMS treatment. rTMS was delivered using the MagPro 100 system (MagVenture, Copenhagen, Denmark) with a figure-8 coil (Coil B658; external wing diameter, 90 mm) placed over F3 (International 10/20 EEG system) to target the left DLPFC. The resting motor threshold (RMT) was defined as the minimum stimulus intensity that produces a motor-evoked potential exceeding 50 μV in a minimum of five of ten trials in the relaxed right abductor pollicis brevis. All patients were treated with 1,600 pulses per session at 10 Hz (1-s trains, 10-s intertrain interval) with 100% RMT intensity. The treatment included 20 sessions of rTMS for 2 weeks [two sessions per day (one in the morning and the other in the afternoon) with approximately 4–6 h between the two sessions; sessions were administered for 5 days per week]. The controls did not receive the RMT examination or any rTMS session.

### Behavioral Assessment

For behavioral assessments, patients completed neuropsychologic tests, including the MoCA and 24 items-HAMD, on the day before rTMS and on the day after completing the last rTMS session. The same trained neurologist conducted behavioral evaluations of each patient to minimize variability and inter-rater bias.

### EEG Recordings and Preprocessing

Resting-state EEGs were recorded on the day before rTMS and on the day after completing the last rTMS session. An eyes-closed resting-state EEG was recorded for 8 min by using a BrainAmp DC amplifier (Brain Products, Munich, Germany) with a 64-channel EEG system. Participants were asked to remain still and relaxed during the EEG recording. The sampling rate was maintained at 5,000 Hz, and electrode impedances were maintained below 5 kΩ by applying the conductive gel. FC_*z*_ was the reference electrode, and AF_*z*_ was the ground electrode during the EEG recording. EEG data were preprocessed offline in MATLAB (R2018a; The MathWorks Inc., Natick, MA, United States) by using a custom script based on EEGLAB (Delorme and Makeig, 2004). The EEG preprocessing steps were: (a) removing typical artifacts by visual inspection; (b) down-sampling to 250 Hz; (c) notch filtering (48–52 Hz) to remove 50 Hz line noise and its harmonics; (d) bandpass filtering between 1 and 45 Hz using a zero-phase finite impulse response filter; (e) rejecting bad channels and spherically interpolating rejected channels; (f) the signals were then segmented into 2-s; (g) removing remaining artifacts by independent components analysis, including ocular, heartbeat, and high-frequency persistent muscle artifacts, and (h) re-referencing to the common average.

### Source-Level PEC Calculation

The estimation of PEC between orthogonalized signals at the source-level was calculated as described in a previous study (Zhang et al., 2020). Source localization was performed via custom scripts and the publicly available toolbox Brainstorm (Tadel et al., 2011). Rotating dipoles at 3003 vertices on the cortical surface were generated. Pearson’s correlation coefficients between the log-transformed power envelopes were thereafter obtained for each pair of vertices. Functional connectivity was calculated among 31 brain regions in the Montreal Neurological Institute space, derived from an independent parcellation of resting-state functional magnetic resonance imaging (fMRI) connectivity using an independent components analysis of 38 healthy individuals derived in a previous study (Chen et al., 2013). For each pair of regions, the Fisher-Z transforms of the Pearson’s correlations of each pair of vertices within each of the regions were averaged to determine the connectivity of that specific pair of regions. A total of 465 pairs of PECs (excluding self-connectivity) was computed for each of the five frequency bands (delta, 1–4 Hz; theta, 5–7 Hz; alpha, 8–12 Hz; beta, 13–30 Hz; and gamma, 31–45 Hz).

### Statistical Analyses

Statistical analyses were conducted using SPSS version 11.0 (SPSS, Inc., Chicago, IL, United States) and MATLAB (The MathWorks Inc.). Normality was checked using the Shapiro–Wilk test. Age, years of education and behavioral measurements (i.e., MoCA and HAMD) were compared between the two patient groups and the control group using one-way analysis of variance. Bonferroni correction was used for *post-hoc* analysis. Sexes were compared using the Fisher’s exact test. Duration of illness and RMT between the two patient groups were compared using the independent samples *t*-test. Behavioral measurements obtained pre- and post-rTMS treatment were compared using the paired *t*-test or the Wilcoxon matched-pairs signed ranks test. Treatment effect sizes were calculated using Cohen’s d (Behl et al., 2014). Correlation analysis between the MoCA and HAMD at baseline was conducted using Pearson’s correlation. Network-based statistics (NBS) were used to evaluate clinical group differences in the functional connectivity matrices with revealing a statistically significant network, using the NBS toolbox (Zalesky et al., 2010). The average connectivity strength in the significant network was calculated, and its changes from pre- to post-rTMS was correlated with the changes of MoCA using Pearson’s correlation. The sum of the number of NBS survived edges (survived edges means edges composing the significant network in the comparison between groups using NBS) for each node was computed. The three-dimensional brain network differences were visualized using the BrainNet Viewer toolbox (National Key Laboratory of Cognitive Neuroscience and Learning, Beijing Normal University, Beijing, China) (Xia et al., 2013).

## Results

### Participants

Both patients and controls successfully completed all EEG examinations and behavioral assessments. All patients tolerated rTMS well with no reports of adverse effects. No significant differences were found between the two-group patients and controls in age (*F* = 3.25, *p* = 0.06), years of education (*F* = 0.15, *p* = 0.06), or sex (*p* = 0.90, Fisher’s exact test). The MoCA scores ranged from 10–17 for the moderate group and 18–28 for the mild group. No significant difference was found between the mild and moderate cognitive impairment groups in terms of the RMT [*t*(21) = 0.35, *p* = 0.72]. There were significant differences between the patient groups and the control group in HAMD (*F* = 5.1, *p* = 0.01). *Post-hoc* test showed a higher HAMD score in the mild and moderate groups than in controls (mild vs. control, *p* = 0.03; moderate vs. control, *p* = 0.03), and no significant differences between the mild and moderate group (*p* > 0.99). No correlation was found between the MoCA and HAMD scores at baseline in the mild (*p* = 0.71, *r* = 0.12) or moderate groups (*p* = 0.39, *r* = 0.26).

### Abnormal Functional Connectivity in AD Pre-rTMS

In general, NBS identified a network of stronger connectivity in the delta and theta band across the whole brain separately, and a network of weaker connectivity in the beta band in the moderate group, compared to the control or mild group at baseline. In the alpha and gamma band, no significant difference existed between the moderate group and the other two groups. No significant difference existed between the mild and control group at any frequency band. The abnormal networks identified in AD patients are as follows.

**FIGURE 1:**
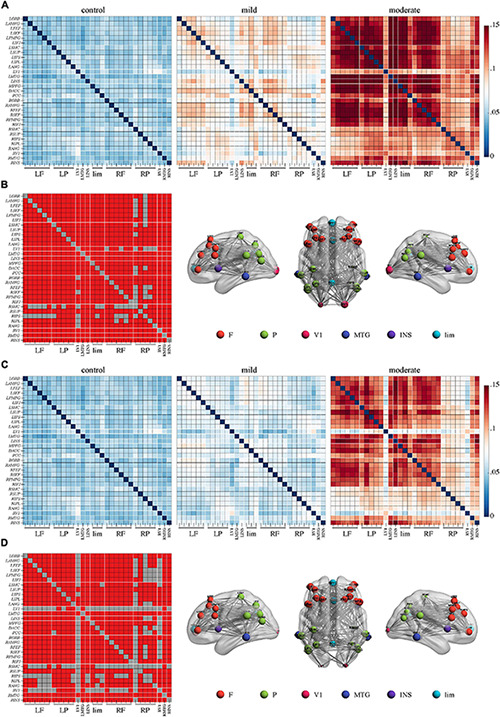
Higher connectivity in the delta band in Alzheimer’s disease patients with moderate cognitive impairment before and after repetitive transcranial magnetic stimulation (rTMS). **(A)** Power envelope connectivity matrices of the control, mild, and moderate groups pre-rTMS. **(B)** The network-based statistics results of moderate group > control group pre-rTMS. **(C)** Power envelope connectivity matrices of the control, mild, and moderate groups post-rTMS. **(D)** The network-based statistics results of moderate group > control group post-rTMS. In **(B,D)**, the connectivity difference matrix (left panel) and its three-dimensional visualization (right panel) are shown. In the matrix, the red squares indicate the survived edges from the network-based statistics analysis. The size of the node represents its sum of the survived edges; the larger the node, the greater the sum, and vice versa. The three-dimensional visualization shows the left, up, and right views of the brain. For all figures (“L” and “R” in front of certain abbreviations (e.g., ORB, AMFG, FEF) means “left” and “right,” respectively): ORB, orbitofrontal cortex; AMFG, anterior division of middle frontal gyrus; FEF, frontal eye field; SEF, supplementary eye field; PMFG, posterior division of middle frontal gyrus; IFJ, inferior frontal junction; SMC, somatosensory cortex; SUP, supramarginal gyrus; IPS, intraparietal sulcus; IPL, inferior parietal lobule; ANG, angular gyrus; V1, primary visual cortex; MTG, middle temporal gyrus; INS, insular cortex; MPFC, medial prefrontal cortex; DACC, dorsal anterior cingulate cortex; PCC, posterior cingulate cortex. F, frontal cortex; P, parietal cortex; lim, limbic lobe.

In the **delta** band, a comparison of PEC between the moderate group and controls by NBS yielded a network of 31 nodes and 433 edges; the connectivity strength was higher in moderate group patients (Figures 1A,B, *p* < 0.05, Bonferroni-corrected). Regions in the bilateral frontal, parietal, temporal, occipital, insular, and limbic lobes and nearly all edges between them were involved in this network. There was no significant difference between the mild group and the control or moderate group.

**FIGURE 2:**
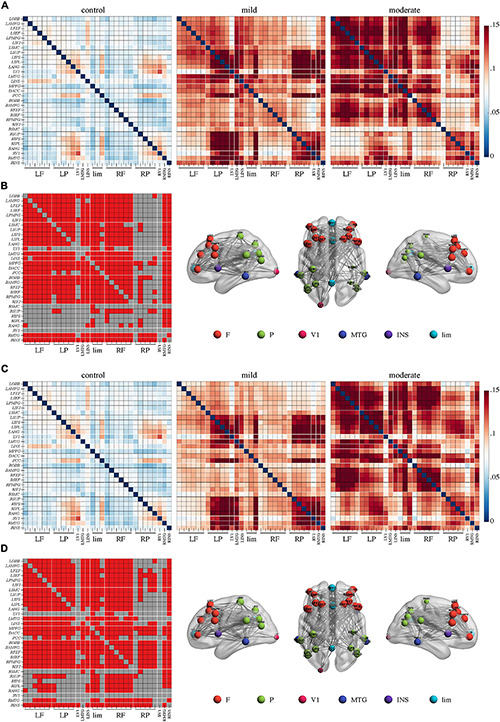
Higher connectivity in the theta band in Alzheimer’s disease patients with moderate cognitive impairment before and after repetitive transcranial magnetic stimulation (rTMS). **(A)** Power envelope connectivity matrices of the control, mild, and moderate groups pre-rTMS. **(B)** The network-based statistics results of moderate group > control group pre-rTMS. **(C)** Power envelope connectivity matrices of the control, mild, and moderate groups post-rTMS. **(D)** The network-based statistics results of moderate group > control group post-rTMS. In **(B,D)**, the connectivity difference matrix (left panel) and its three-dimensional visualization (right panel) are shown. The three-dimensional visualization shows the left, up, and right views of the brain. The meaning of the red squares, size of node, and all abbreviations are the same as in Figure 1.

In the **theta** band, a network of 31 nodes and 307 edges with a higher PEC was identified by NBS in the moderate group, compared to controls (Figures 2A,B, *p* < 0.05, Bonferroni-corrected). Regions across the whole brain were involved in this network. Regions in right posterior areas, such as the right sensorimotor cortex, supramarginal gyrus, intraparietal sulcus, inferior parietal lobule, angular gyrus, and primary visual cortex, had a relatively lower sum of survived edges. A comparison between the mild group and the control or moderate group did not reveal any significant difference (*p* > 0.05), though an increase tendency of the connectivity strength than the control was observed in the mild group.

**FIGURE 3:**
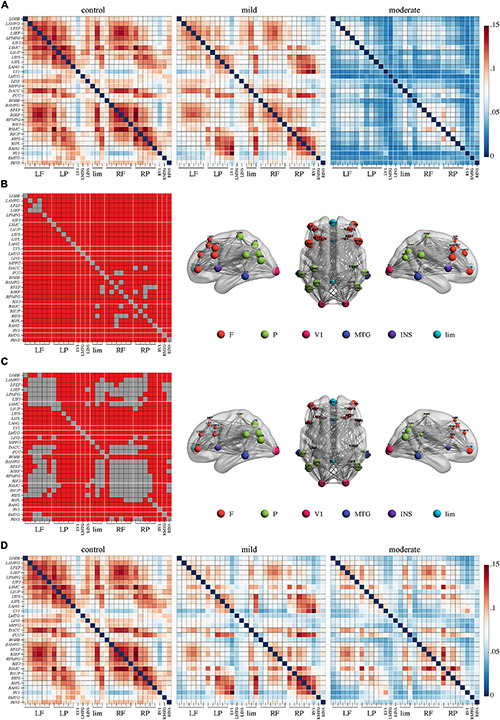
Weaker connectivity in the beta frequency band in Alzheimer’s disease patients with moderate cognitive impairment before repetitive transcranial magnetic stimulation (rTMS). **(A)** Power envelope connectivity matrices of the control, mild, and moderate groups pre-rTMS. **(B)** The network-based statistics results of control group > moderate group pre-rTMS. Connectivity difference matrix (left panel) and its three-dimensional visualization (right panel) are shown. **(C)** The network-based statistics results of mild group > moderate group pre-rTMS. **(D)** Power envelope connectivity matrices of the control, mild, and moderate groups pre-rTMS. In **(B,C)**, the connectivity difference matrix (left panel) and its three-dimensional visualization (right panel) are shown. The three-dimensional visualization shows the left, up, and right views of the brain. The meaning of the red squares, size of node, and all abbreviations are the same as in Figure 1.

In the **beta** band, the comparison between the moderate and control group yielded a network of 31 nodes and 453 edges with a significantly lower PEC strength in the moderate group by NBS (Figures 3A,B, *p* < 0.05, Bonferroni-corrected). Regions across the whole brain and nearly all edges between them were involved in this network. Meanwhile, the comparison between the moderate and mild groups revealed a network of 31 nodes and 335 edges with lower connectivity in the moderate group (Figures 3A,C, *p* < 0.05, Bonferroni-corrected). Regions in the bilateral frontal lobes such as the bilateral anterior division of middle frontal gyrus, frontal eye field, supplementary eye field, posterior division of middle frontal gyrus, and inferior frontal junction had a lower sum of survived edges. No significant difference was found in the comparison between the mild and control groups.

### Effects of rTMS on Clinical Performance

General cognitive function improved after rTMS treatment because the MoCA scores significantly increased after rTMS treatment in the mild group [pre-rTMS, 23.2 ± 3.7; post-rTMS, 25.5 ± 3.9; *t*(11) = 3.44; *p* = 0.01; Cohen’s d = 0.99] and in the moderate group [pre-rTMS, 13.4 ± 2; post-rTMS, 17.2 ± 3.31; t(10) = 3.37; *p* = 0.01; Cohen’s *d* = 1.01]. Depressive symptoms were not ameliorated because no statistical differences were found between the pre- and post-rTMS HAMD scores in the mild group [pre-rTMS, 9.8 ± 3.9; post-rTMS, 5.9 ± 4.8; *t*(11) = 2.03; *p* = 0.07] or in the moderate group [pre-rTMS, 9.9 ± 6.1; post-rTMS, 7.5 ± 5.4; *t*(10) = 1.39; *p* = 0.20].

### Effects of rTMS on Functional Connectivity

After rTMS treatment, stronger PEC networks in the delta and theta bands across the whole brain were persistently identified in the moderate group compared to the control group (*p* < 0.05, Bonferroni-corrected). The network in the delta band involved 31 nodes and 379 edges (Figures 1C,D). The network in the theta band involved 31 nodes and 323 edges (Figures 2C,D). No significant difference was revealed in the beta band between the moderate group and the control group after rTMS (Figure 3D). No significant difference was found between the PEC of the mild group post-rTMS and the control group.

**FIGURE 4:**
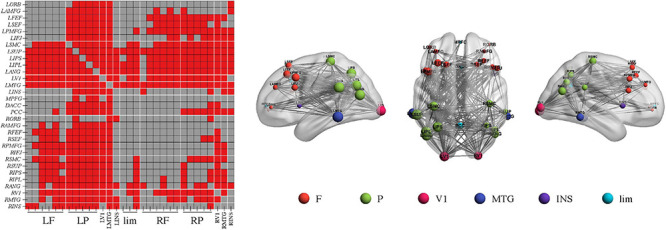
Repetitive transcranial magnetic stimulation (rTMS) improves power envelope connectivity in the beta band in Alzheimer’s disease patients with moderate cognitive impairment. The network-based statistics results of post-rTMS > pre-rTMS in moderate group was shown. Connectivity difference matrix (left panel) and its three-dimensional visualization (right panel) are shown. The three-dimensional visualization shows the left, up, and right views of the brain. The meaning of the red squares, size of node, and all abbreviations are the same as in Figure 1.

Importantly, NBS identified one network in the beta band in the comparison of the PEC between pre- and post-rTMS in the moderate group (Figure 4, *p* < 0.05, Bonferroni-corrected). The network of 31 nodes and 265 edges was yielded with increased PEC after rTMS treatment, and nodes in the left parietal-occipital regions such as the left somatosensory cortex, supramarginal gyrus, intraparietal sulcus, inferior parietal lobule, angular gyrus, primary visual cortex, and the left middle temporal gyrus had a relatively higher sum of survived edges. Moreover, the increase of the connectivity strength in this network was positively correlated with the increase of MoCA scores after rTMS treatment (*r* = 0.75, *p* = 0.01). No significant difference in PEC was found between pre- and post-rTMS in the delta (Figures 1A,C, *p* > 0.05) and theta (Figures 2A,C, *p* > 0.05) bands, though a decrease tendency was observed in the delta band. In the mild group, no significant difference was found in a comparison of the PEC between the pre- and post-rTMS PEC in these frequency bands.

## Discussion

This study was an investigation of how DLPFC rTMS exerts its effects of improving cognition by altering electroencephalographic functional connectivity in AD. Compared to controls, AD patients with moderate cognitive impairment had increased connectivity in the delta and theta bands but decreased connectivity in the beta band. With rTMS treatment, beta connectivity increased along with an improvement in cognitive function in patients with moderate cognitive impairment, whereas there were no significant alterations of the connectivity in the delta and theta bands. However, these baseline abnormalities and rTMS modulation effects were not found in AD patients with mild cognitive decline.

According to the diagnostic guidelines of the International Working Group and the US National Institute on Aging Alzheimer’s Association (NIA-AA), AD status is associated with β-amyloid (Aβ) pathology, tau pathology, other biomarkers of neurodegeneration such as fluorodeoxyglucose positron emission tomography (Dubois et al., 2014; Jack et al., 2018). Although these methodologies refine the pathophysiology and staging of AD, their uses are limited in the general clinical practice because of the invasiveness or cost. In this regard, other non-invasive and cost-effective modalities associated with disease stage and cognitive symptoms, such as EEG as well as motor assessment, should be stated as integral components in the clinical work-up (Voglein et al., 2019; Babiloni et al., 2020).

In EEG studies, a lower beta coherence in patients with AD than in controls has been consistently reported (Adler et al., 2003; Pogarell et al., 2005; Fonseca et al., 2011). Decreased connectivity in the beta rhythm has been cross-validated by alternative techniques such as phase lagged index and synchronization likelihood (Stam et al., 2005; Babiloni et al., 2020). The present study displayed significantly decreased beta PEC in patients with moderate cognitive impairment, but not in patients with mild cognitive impairment, compared to controls. In contrast to the coherence and phase lagged index, PEC measures the correlation between slowly fluctuating power envelope signals, independent of the signal phase (Hipp et al., 2012). PEC is more reliable than the phase lagged index for examining AD neurophysiology by EEG (Briels et al., 2020b). An MEG study, which enrolled AD patients with a Mini-Mental State Examination score ≥ 19, reported disrupted beta PEC in AD patients (Koelewijn et al., 2017). These differences between the MEG and EEG findings might be because of the different signal-to-noise ratios of these two modalities, or because patients with different severity levels of cognitive dysfunction and different measurement methods were used in different studies. Of importance, the effects of rTMS on the functional connectivity were limited to the beta band but not the delta and theta bands in this study, and the more increase of beta connectivity strength, the more increase of MoCA scores after rTMS. Similarly in major depressive disorder and bipolar disorder, rTMS to the left prefrontal cortex at 10 Hz increased EEG functional connectivity in the beta band (Kito et al., 2017; Zuchowicz et al., 2018).

The functions of beta oscillation are not well understood at present. Beta activity has traditionally been associated with sensorimotor processing, and recent research has suggested its role in various cognitive functions (Spitzer and Haegens, 2017). During working memory tasks, the frontal cortex is synchronized with the parietal, temporal, or occipital cortices via beta oscillations (Salazar et al., 2012; Mendoza-Halliday et al., 2014). Beta activity may also be related to maintaining the current cognitive state (Engel and Fries, 2010). It has been found that the generation of the beta rhythm is partially linearly dependent on the concentration of inhibitory γ-aminobutyric acid in the brain (Gaetz et al., 2011). Increasing evidence has emerged to disclose the dysfunction of γ-aminobutyric acid-ergic signaling system are implicated in the pathophysiology of AD (Li et al., 2016). The elevated prefrontal cortex γ-aminobutyric acid had been implicated in the mechanism of action of high-frequency rTMS (Dubin et al., 2016). Thus, high-frequency rTMS may modulate the beta rhythm by influencing the activity of γ-aminobutyric acid. Though no significant correlation between the change of beta connectivity and the alteration of the depressive symptom after rTMS in major depressive disorder (Kito et al., 2017), here we also found that the increase of the beta connectivity was positively correlated with the improvement of general cognitive function with rTMS treatment. Perhaps the concentration of γ-aminobutyric acid which contributed to the generation of beta rhythm and the cognitive function was improved by rTMS. Though further research is required, these findings indicate the role of beta connectivity in cognitive impairment and may be a mechanism underpinning physiological changes to the human cortex after rTMS.

Delta and theta oscillations have been proved to be involved in multiple cognitive processes (Harmony, 2013; Solomon et al., 2017). However, variable results of altered connectivity in the delta and theta bands have been reported in AD, with findings of the connectivity being globally or locally increased or decreased (Babiloni et al., 2016; Gouw et al., 2017). The current EEG study showed that AD patients with mild cognitive impairment had no significant alteration in delta and theta PEC, while patients with moderate cognitive impairment had higher delta and theta PEC than controls. The MEG study mentioned above also reported no significant abnormalities in the delta and theta PEC in patients with a Mini-Mental State Examination score ≥ 19 (Koelewijn et al., 2017). The effects of rTMS on the delta and theta bands are also inconclusive with lack of enough evidence. As the two above mentioned studies, although both observed increase of beta connectivity after 20 sessions 10 Hz rTMS, one reported no significant changes of lagged phase synchronization in delta and theta bands in treatment-resistant depression (Kito et al., 2017), the other found the different change tendency of phase locking value in rTMS responder or non-responders (Zuchowicz et al., 2018). The different functional connectivity measures and frequency band can influence the EEG studied outcome. It was demonstrated that the reproducible effects of orthogonalized amplitude envelope correlation were found in the alpha and beta bands, while phase-based measures in the theta band (Briels et al., 2020a). The effects of 10 Hz rTMS on the slow-wave connectivity deserve further studies.

The DLPFC is one of the most popular targets in cognition rTMS studies. It lies primarily in Brodmann areas 46, 9, and 9/46 and is involved in various cognitive functions such as memory, attention, and execution (Carlen, 2017). Anatomical evidence derived from non-human primate studies using direct anterograde and retrograde tracing has defined local projections within the prefrontal cortex in conjunction with long connection pathways between the prefrontal cortex and the parietal, occipital, temporal, insular, and limbic lobe (Yeterian et al., 2012). Neuroimaging studies using fMRI and diffusion tensor imaging have shown equivalent connectivity patterns in the human and non-human primate brain (Thiebaut de Schotten et al., 2012), which shows that use of non-human primate data to understand human brain connectivity is reasonable. In fMRI studies (Beynel et al., 2020), rTMS-induced functional connectivity changes occurred within the stimulated site locally and across the whole-brain network. The current EEG study indicated that rTMS over the left DLPFC significantly improved beta connectivity at a whole-brain level, especially in the left parietal-occipital regions. Based on the anatomical basis of brain connectivity and the rTMS feature of influencing areas distant from the stimulation site, the significant changes in the left parietal-occipital cortex connectivity after left DLPFC rTMS are understandable. Moreover, the lateral parietal cortex has projections to the hippocampus and an important role in memory (Wagner et al., 2005). High-frequency rTMS over the left parietal cortex improved associative memory performance and concomitantly increased functional connectivity between the hippocampus and the left lateral parietal cortex, as well as the hippocampal intrinsic connectivity networks (Wang et al., 2014). Whether targeting the left parietal cortex would be alternative or complementary to the left DLPFC rTMS for improving cognitive functions deserves thorough investigation.

In addition, the DLPFC is a prominent region where cognition and emotion interact (Ray and Zald, 2012). The stimulation protocol of 10-Hz rTMS over the left DLPFC is primally a strategy for treating depression. Whether the effects of rTMS on cognitive function in this study were just an appendage of improved depressive symptoms should not be ignored. However, our results showed no significant remission of depressive emotion in this cohort of patients with AD who presented with some depressive symptoms in the mild and the moderate group. A previous study (Ahmed et al., 2012) reported that 20-Hz rTMS over the bilateral DLPFC, one session per day for 5 consecutive days, improved both Mini Mental State Examination and the Geriatric Depression Scale scores in AD. This finding may differ from that of this study because of the variation in the stimulation protocol or the distinct measurements used.

rTMS effects may rely on the brain status, which may differ from the intrinsic ability to compensate or restore impaired neuronal function. Previous investigators have reported that rTMS improves action, but not object naming accuracy, in mild AD, and that it improves both features in moderate/severe AD (Cotelli et al., 2008). This finding suggests that rTMS has distinct effects for different states of cognitive impairment. In this study, although general cognition was enhanced in the mild and moderate groups after rTMS, functional connectivity was only altered in the moderate group. The unchanged connection in the mild group may derive from its relative normality at baseline. Of course, more sensitive EEG biomarkers for distinguishing between AD patients with mild cognitive impairment and healthy controls should be defined and used in future studies. The correlation between the severity of cognitive impairment and PEC should be examined using a larger dataset. In addition, we noticed that, because the MoCA tests were performed in the same manner before and after rTMS treatment in this study, some patients may have learned or practiced tasks to perform better over time, as previously reported (Rutherford et al., 2015). This factor may be more likely and may more easily occur among patients with mild cognitive decline. If the cognitive improvement resulted from practice rather than from an rTMS effect, then more objective and sensitive tools such as EEG features should potentially be used complementarily for assessing rTMS efficacy, as in AD clinical trials of drugs (Rodriguez et al., 2002; Adler et al., 2004; Scheltens et al., 2018).

Some limitations existed in this study. The lack of a sham stimulation group prevented us from determining whether some treatment effects were placebo-related. However, evidence from previous studies supports the treatment efficacy of rTMS for cognitive enhancement in people with AD (Chou et al., 2020). In addition, the purpose of this study was to gain insight into the role of functional connectivity as a mechanism facilitating improvements in cognitive function with rTMS. A further limitation is that the diagnostic criteria for probable AD were primarily based on clinical manifestations and not on biological markers of the cerebrospinal fluid because of the lack of patient agreement and unavailability of positron emission tomography at our hospital. The use of same version of MoCA for pre- and post-rTMS with interval of 2 weeks was another limitation. In addition, we used 31 brain regions derived from independent component analysis parcellation of fMRI connectivity which might introduce an overlapping effect on the EEG signal. Moreover, the sample size was small, and patients aged 50–75 years were included, covering the early onset and the late-onset AD which might involve different mechanisms. Pathological markers for AD diagnosis, comprehensive neurological measurements and larger sample sizes separating early-onset and the late-onset AD should be considered in future research.

## Conclusion

We found that AD patients with moderate cognitive impairment had increased delta and theta connectivity and decreased beta connectivity, compared to healthy controls. rTMS treatment led to a significant clinical improvement in cognitive function and increased functional connectivity in the beta band. The present results indicated the network level effect of the rTMS in improving cognitive function in AD and inform future efforts to optimize rTMS treatment protocols, based on electroencephalographic functional connectivity. Furthermore, beyond the cognitive enhancements found in this study, we propose future research should consider motor symptoms which are influenced by cognition and could be improved by neuromodulation techniques in AD.

## Data Availability Statement

The raw data supporting the conclusions of this article will be made available by the authors, without undue reservation.

## Ethics Statement

The studies involving human participants were reviewed and approved by Institutional Review Board of Shenzhen People’s Hospital (Shenzhen, China). The patients/participants provided their written informed consent to participate in this study.

## Author Contributions

YG: conceptualization, methodology, supervision, validation, writing—reviewing, and editing. GD: writing—original draft preparation, software, and formal analysis. BH: methodology, writing—reviewing, and editing. XSu: investigation, writing—reviewing, and editing. NY: writing—reviewing and editing. SC, HR, XSh, MC, SZ, and XL: investigation, writing—reviewing, and editing. All authors contributed to the article and approved the submitted version.

## Conflict of Interest

The authors declare that the research was conducted in the absence of any commercial or financial relationships that could be construed as a potential conflict of interest.

## Footnotes

1 https://www.mocatest.org/faq/
